# Long-Term Outcome of Elderly Patients with Severe Aortic Stenosis Undergoing a Tailored Interventional Treatment Using Frailty-Based Management: Beyond the Five-Year Horizon

**DOI:** 10.3390/jpm14121164

**Published:** 2024-12-21

**Authors:** Augusto Esposito, Ilenia Foffa, Paola Quadrelli, Luca Bastiani, Cecilia Vecoli, Serena Del Turco, Sergio Berti, Annamaria Mazzone

**Affiliations:** 1 Cardiology Unit, Ospedale del Cuore, Fondazione Toscana “G. Monasterio”, 54100 Massa, Italy; ilenia.foffa@cnr.it (I.F.); quadrelli@monasterio.it (P.Q.); luca.bastiani@cnr.it (L.B.); cecilia.vecoli@cnr.it (C.V.); sergio.berti@ftgm.it (S.B.); 2 Institute of Clinical Physiology, National Research Council, 54100 Massa, Italy; serena.delturco@cnr.it

**Keywords:** aortic stenosis, elderly patients, tailored interventional treatment, frailty-based management, outcome, long-term follow-up

## Abstract

**Background:** Elderly patients with severe aortic stenosis (AS) need individualized decision-making in their management in order to benefit in terms of survival and improvement of quality of life. Frailty, a common condition in elderly patients, needs to be considered when weighing treatment options. **Aim:** We aimed to evaluate outcomes including survival and functional parameters according to disability criteria at six years of follow-up in an older population treated for severe AS using a frailty-based management. **Methods:** We evaluated data derived from a pilot clinical project involving elderly patients with severe AS referred to a tailored management based on classification by Fried’s score into pre-frail, early frail, and frail and a multidimensional geriatric assessment. A Frailty, Inflammation, Malnutrition, and Sarcopenia (FIMS) score was used to predict the risk of mortality at six years of follow-up. Functional status was evaluated by telephonic interview. **Results:** At six years of follow-up, we found a survival rate of 40%. It was higher in the pre-frail patients (long rank < 0.001) and in the patients who underwent TAVR treatment (long rank < 0.001). The cut-off FIMS score value of ≥1.28 was an independent determinant associated with a higher risk of mortality at six years of follow-up (HR 2.91; CI 95% 1.7–5.1; *p*-value 0.001). We found a moderate increase of disability levels, malnutrition status, comorbidities, and number of drugs, but none of them self-reported advanced NYHA class III–IV heart failure. **Conclusion:** An accurate clinical–instrumental and functional geriatric evaluation in an elderly population with AS is required for a non-futile interventional treatment in terms of survival and functional status even in long-term follow-up.

## 1. Introduction

Aortic stenosis (AS), particularly severe aortic stenosis, will increase in incidence as the average age increases [1,2]. The treatment of elderly patients with severe aortic stenosis requires appropriate individualized decision-making in patient management. Transcatheter aortic valve replacement (TAVR) has emerged as the primary choice in elderly patients with symptomatic severe AS when surgical and percutaneous options are both feasible, independently of their surgical risk profile [3,4]. Therefore, a general assessment of the elderly patient appears to be necessary, considering the parameters related to cognitive and functional impairment, polypharmacy, malnutrition, chronic inflammatory status, sarcopenia, and frailty [5]. It is now known that the benefit of TAVR in frail patients is poor or absent [6,7]. Therefore, a multidisciplinary evaluation appears mandatory to estimate the real expected benefit in terms of survival and improvement of quality of life to define a tailor-made treatment pathway for aortic pathology in the elderly [7,8,9]. An innovative but consolidated treatment for patients previously untreated due to high or prohibitive surgical risk is represented by transcatheter aortic valve replacement (TAVR), but the selection of patients who can benefit from this procedure is necessary and represents a challenge [8,9]. In a complex patient population, the integration and identification of relevant prognostic information is crucial. Typically, a multidisciplinary team assesses clinical and operative risk using scores such as the EuroSCORE II and the Society of Thoracic Surgeons (STS) score. In the elderly population, frailty is known to be associated with increased mortality and disability rates following TAVR, so a preoperative assessment of the degree of frailty in conjunction with common traditional risk scores is useful for predicting postoperative outcomes [10]. Several studies have shown that frailty assessment in addition to the usual predictive risk models improves short- and medium-term mortality rates after TAVR [10,11,12]. However, long-term data on clinical outcomes after TAVR are limited. In our previous articles, we showed that pre- and early-frailty patients seem to be ideal candidates for TAVR/SAVR, with high mid-term survival. In addition, we showed that the application of multidisciplinary preoperative evaluation aimed at “management tailored on frailty” could lead to better clinical outcomes in the elderly population. In contrast, advanced frailty status with high comorbidity and disability is strongly associated with mortality regardless of interventional or medical treatment, whether futile or palliative. [13]. In addition, we approved and tested a user-friendly score based on clinical chemistry parameters that can identify frailty and predict mortality in patients with severe aortic stenosis (AS) 20 months into follow-up for overall and cardiovascular mortality [5,13]. This score can be a useful guide in the care pathway related to elderly patients with severe aortic stenosis.

The aim of our study is to evaluate outcomes such as survival and quality of life according to disability criteria at a longer time (six years) of follow-up in the same older population in relation to frailty degree and treatment of AS. In the survivors, we aimed to assess the evolution of functional parameters such as nutritional status, disabilities, comorbidities, and polypharmacy as indicators of post-discharge quality of life.

## 2. Methods

Data derived from a pilot clinical study on 109 elderly patients (median age ± SD, 83.3 ± 5.5; 32% males) with severe AS were evaluated. Fried criteria and geriatric, clinical, and surgical features were used to perform a tailored management among surgical aortic valve replacement (SAVR), transcatheter aortic valve replacement (TAVR), balloon aortic valvuloplasty (BAV), and medical therapy (MT) [13]. A cut-off point of ≥1.28 for the Frailty, Inflammation, Malnutrition, and Sarcopenia score (FIMS score) based on routine clinical chemistry parameters, previously valued in the structural equation model (SEM) [5], was used to predict the risk of mortality at six years of follow-up in the elderly population with severe aortic stenosis. Survival at six years was assessed in all patients by telephonic follow-up or review of electronic medical records. In particular, the survivor group was called via phone by a member of the research team to obtain information about nutritional status (MNA), cognitive disorders, polypharmacy, functional limitation, and disability in performing specific basic activities of daily living (BADL) and instrumental activities of daily living (IADL). Mostly patients were not able to perform a visit at our center. The interview was always conducted directly with the patient and preferably in the presence of a family member or caregiver who knew the patient well. The study was approved by the local Ethics Committee (Comitato Etico di Area Vasta Nord Ovest [CEAVNO] No. 22239/2022).

## 3. Statistical Analysis

All statistical analyses were completed using SPSS Version 24 (IBM Corp, Armonk, NY, USA), and significance was set at *p* < 0.05. Categorical variables were expressed as percentages, while continuous variables were expressed as a mean ± standard deviation (SD) or interquartile range (IQR). For comparison between categorical and nominal variables, the Pearson chi-square test and the Fisher’s exact test were used, while to compare continuous data in independent groups, Student’s *t*-test or the Mann–Whitney U test was performed. For the comparison of geriatric functional assessment between baseline and follow-up, the non-parametric Wilcoxon test was performed. Kaplan–Meier and long-rank tests were used to study survival in both the three levels of frailty and the treatment of patients. Univariate Cox regression analysis was performed to explore the association between the single covariates and mortality. Lastly, significant factors were tested again using multivariate Cox regression, with stepwise backward conditional elimination of non-significant factors in the model predicting the risk of mortality at six years of follow-up by testing the covariate of Frailty, Inflammation, Malnutrition, and Sarcopenia score (FIMS score ≥ 1.28).

## 4. Results

One hundred and nine elderly patients with symptomatic severe AS undergoing a multidisciplinary evaluation for a tailored treatment for AS were divided according to Fried frailty phenotype into pre-frail (39.5%), early frail (25.5%), and frail (35%) groups. No patient had a robust result. Patients undergoing SAVR and TAVR in the pre-frail group were 19% and 81% of the group, respectively. In the early frail group of patients, 75% were treated by TAVR and 25% by BAV. In the frail group, only 8% of the patients were addressed for TAVR because they successively needed urgent surgical treatment for neoplastic disease; 55% were addressed for BAV, and 37% of patients were treated with MT. At six years of follow up, we found 44 (40%) survivor patients, 31% of whom are male patients. Clinical and demographic data of the survivor and non-survivor patients are shown in Table 1.

Most of the survivor patients underwent SAVR and TAVR treatment. In the BAV group, in four patients, balloon aortic valvuloplasty was performed as bridge to TAVR. Phone follow-up was performed only for 32 patients. Twelve patients were not easily accessible by telephone because of an unknown phone number or their phone being disconnected and subsequently were not included in the data analysis. However, non-responders were traced through computerized hospital reporting systems and were categorized as survivors. In Table 2 we reported the geriatric functional assessment at the time of the phone follow-up with respect to the basal evaluation.

**Figure 1:**
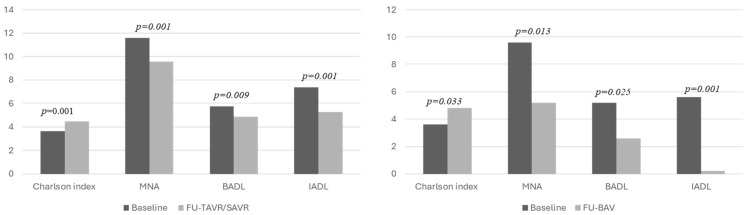
Geriatric functional assessment at follow-up compared to basal evaluation, related to the type of intervention (surgical aortic valve replacement/transcatheter aortic valve replacement and balloon aortic valve).

In particular, six years after treatment, we found a significantly lower Mini Nutritional Assessment (MNA) (11.28 vs. 8.91; *p* < 0.001), BADL (5.66 vs. 4.5, *p* < 0.001), and IADL (7.09 vs. 4.47, *p* < 0.001) scores with respect to the pre-interventional evaluation, as well as a higher Charlson index (3.63 vs. 4.54; *p* < 0.001). The worsening of the geriatric functional assessment scores with respect to the basal evaluation was more evident in patients treated with BAV than in patients treated with TAVR/SAVR (Figure 1).

Interestingly, the survivor patients at six years of follow up were in NYHA class I–II. In addition, in the survival group, we found that four patients (12%) underwent rehospitalization for heart failure; regarding neuropsychological disorders, we found that 46.9% of the patients showed modest depression and 3.1% severe depression.

**Figure 2:**
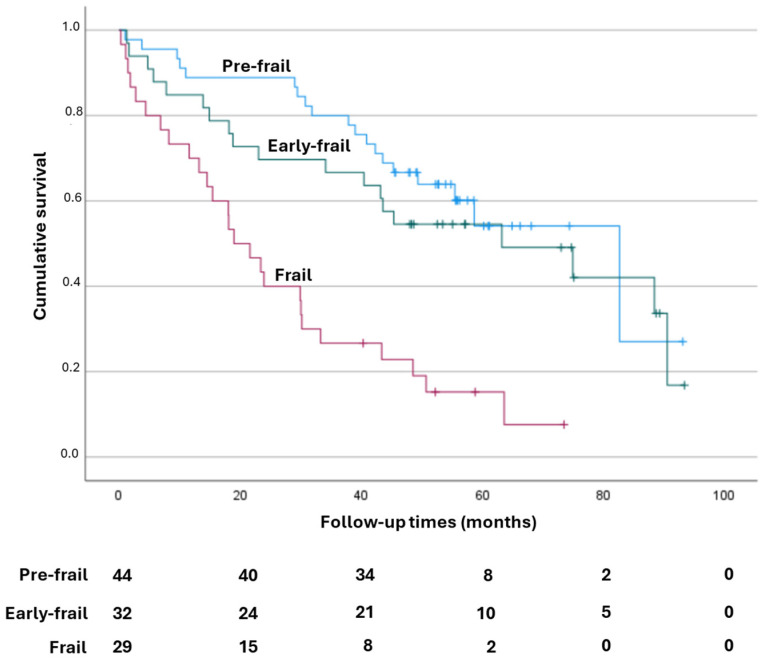
Kaplan–Meier survival curves. The survival rate at 6 years was higher in pre-frail patients, long rank < 0.0001).

Kaplan–Meier analysis showed that, at 6 years of follow-up, the survival rate was higher in the pre-frail patients (long rank < 0.001, Figure 2) in particular, with average survival times of 63.3 months in pre-frail patents, 59.1 months in early frail patients, and 27.5 months in frail patients.

**Figure 3:**
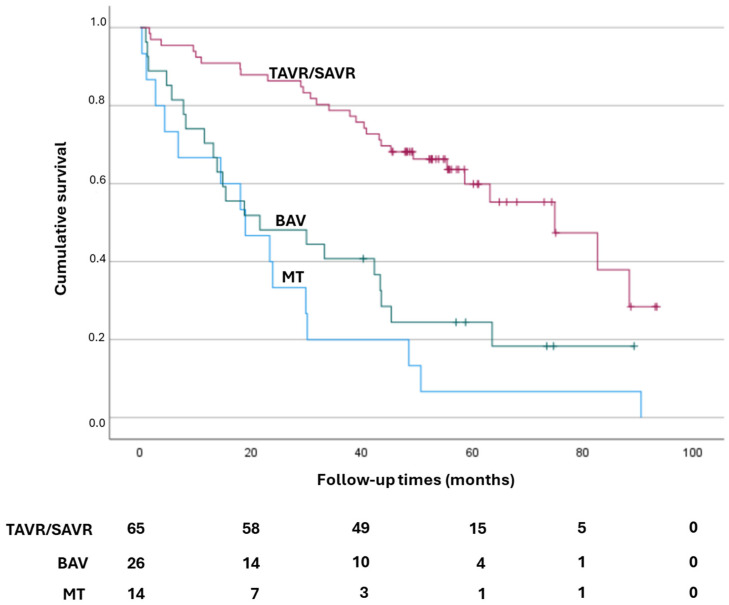
Kaplan–Meier survival curves. The survival rate at 6 years was higher in the patients who underwent TAVR treatment, long rank < 0.0001). SAVR: surgical aortic valve replacement; TAVR: transcatheter aortic valve replacement; BAV: balloon aortic valve; MT: medical therapy.

In addition, we found a higher survival rate in the patients who underwent TAVR treatment (long rank < 0.001, Figure 3), in particular, with the results being 67.6 months for TAVR patients, 34.8 months for BAV patients, and 25.7 months for MT patients.

Regarding FIMS score, based on standard laboratory parameters, at 6 years of follow-up, we found that 70% of died patients met the cut-off FIMS score value of ≥1.28. In particular, deceased patients showed an average value of FIMS score of 2.1, while surviving patients showed a value of 1.0. Univariate Cox regression analysis showed that FIMS, BADL, IADL, frailty degree, and type of treatment were associated with an increased risk of all-cause mortality. Subsequently, the multivariate Cox proportional hazard with stepwise backward conditional elimination of non-significant factors showed that FIMS (hazard ratio, HR (95% CI), 2.1 (1.1–3.7), *p* = 0.003), BADL (HR (95% CI), 0.7 (0.5–0.9), *p* = 0.02), IADL (HR (95% CI), 1.5 (1.2–1.7), *p* = 0.0001), MT treatment ((MT vs. TAVR/SAVR) HR (95% CI), 14.01 (5.8–33.6), *p* = 0.0001), and BAV treatment ((BAV vs. TAVR/SAVR) HR (95% CI), 4.9 (1.2–1.7), *p* = 0.0001) were associated with an increased risk of all-cause death during the 6 years of follow-up in a multivariate model adjusted for sex and age.

## 5. Discussion

Decision-making on transcatheter aortic valve replacement in patients aged 75 years and older is complex. Conventional surgical scores, usually considered in clinical practice, have several limitations in defining risk among candidates for TAVR [8]. This study is the continuation of our two previous ones [5,13] and has as its first objective the prospective evaluation of survival, at a 6-year follow up, of elderly patients with AS treated according to the frailty criterion with interventional therapy (pre-frail/early frail) and valvuloplasty or medical therapy (advanced frailty and acute heart failure) [13]. We confirmed, even at a prolonged follow-up, significant survival of elderly patients with severe AS and initial levels of frailty, who were therefore treated with TAVR/SAVR, compared to advanced frail patients with comorbidities, disabilities, and/or previous events of acute decompensation, who were referred for valvuloplasty and medical therapy. So, we may confirm that “frailty degree-based management” together with clinical, surgical, and anatomical criteria may be crucial to referring elderly patients with severe AS toward an effective and appropriate interventional treatment. Initially, TAVR was planned as a therapeutic strategy in elderly individuals at high risk of surgical valve substitution [10]. Currently, the dynamic advancement of TAVR technology has improved the stratification of elderly patients, taking into account frailty parameters and general geriatric vulnerabilities. A multidisciplinary framework must be employed before performing TAVR procedures, considering geriatric evaluation, both physical and cognitive capabilities, and post-procedural outcomes. In our study, we demonstrated that the selection of SAVR/TAVR for older patients with severe AS and early frail status showed a benefit in terms of survival in the short and long term. Overall, 22% of the 6-year survivors underwent BAV as a bridge surgery for TAVR, which was then performed effectively in 67% of patients. These patients, despite being early frail after an acute episode of heart failure with residual renal insufficiency, had no clinical or functional indications for urgent TAVR/SAVR treatment for AS [14]. Conversely, no patient with advanced frailty, high comorbidity, and disability treated with medical therapy survived 6 years of follow-up. In this population, both interventional and medical treatment of AS are palliative, and the advanced level of frailty is independently associated with global mortality [13]. Frailty is an element of geriatric assessment, so more comprehensive geriatric parameters need to be routinely incorporated into clinical management of complex older cardiovascular patients. In fact, several studies support the role of comprehensive geriatric assessment (CGA) also in cardiovascular disease [7,15,16], using frailty scores that help to estimate the mortality risk and identify patients likely benefit from TAVR/SAVR [9]. In fact, older adults with competing comorbidities and geriatric syndromes have suboptimal quality of life outcomes, highlighting the cumulative vulnerability that persists despite valve replacement [17]. It appears important to identify patients in whom 2-year mortality is very high and for whom TAVR treatment is thereby futile. Several risk scores have been proposed to predict “futile” outcomes [9], but also, a multidisciplinary heart team that includes geriatricians can allow caregivers to optimize care and avoid futile therapeutic interventions in elderly patients, as confirmed by our data. However, for routine clinical use, current frailty scales are too complex. Therefore, simple tools able to assess patients’ frailty and to predict mortality in elderly patients are needed. Shimura et al., using a Japanese multicenter registry to review data of 1215 patients, tested a simple, semiquantitative Clinical Frailty Scale (CFS) for predicting the prognosis of patients who underwent TAVR [18]. The scale focuses on items that are relatively easy to interpret even without specialized training, including mobility, balance, use of walking aids, and the ability to eat, dress, shop, and cook.

In our previous paper, we developed a novel and easy to use FIMS score, based on standard laboratory parameters, that demonstrated a good correlation with the phenotypic Fried score usually used in clinical practice [5]. This score highlighted the impact of malnutrition, inflammation, sarcopenia, and physical frailty on adverse outcomes in elderly patients with severe AS. In fact, physical frailty and sarcopenia share several traits, such as a reduction of lean mass and physical function [19], and malnutrition plays a role in the pathogenesis of both conditions and vice versa [20]. Therefore, given their association with adverse outcomes, the assessments of these three features have become a growing field of interest. It could be important to adopt physical activity and nutritional strategies to modify or reverse the frailty syndrome. In this long-term study, we potentially confirmed the ability of the FIMS score to guide treatment strategy decision-making in elderly patients with severe symptomatic SA, while also confirming the ability of FIMS score to predict mortality in this population. However, future large-scale prospective studies in different aging populations are necessary to confirm the utility of the FIMS score in supporting tailored therapeutic management. Our study showed a survival rate of 40%, and these data are comparable to five-year survival rates ranging from 26% to 46% in various other study cohorts, confirming the importance of tailored treatment based on frailty status in elderly populations with AS [21,22,23,24] and that when TAVR/SAVR treatment is performed in early frail patients, they show a good survival rate. Several clinical and observational studies have demonstrated improvement in functional status after TAVR and SAVR [25]. However, the evidence may be insufficient for tailored decision making and clinical care in older adults with significant heterogeneity in physical condition. In our clinical study, the second but not less important objective was to indicate the health trajectory, in surviving early frail patients treated with TAVR/SAVR while considering their health status, at the time of a long-term follow up. The surviving patients, contacted by telephone at home, showed a moderate but significant increase in disability levels (BADL; IADL) and malnutrition status (MNA), comorbidities (Charlson), and pharmacotherapy among patients treated with BAV. This subgroup of patients included the early frail category who have had a recent episode of acute decompensation with clinical treatment and with the level of frailty worsening, for which treatment with BAV was less invasive as a bridge for a subsequent TAVR. Their systolic function was preserved (EF 51%) but moderately reduced compared to the patients without acute heart failure events (60%) [14].

None of the surviving patients self-reported as being in advanced NYHA class III–IV. Of them, only 12% required rehospitalization for acute heart decompensation, and most patients no showed signs of dementia and/or depression. The detection of the functional status and therefore the quality of life after aortic valve replacement is important. Our data agree with Kim DH et al., suggesting that functional deterioration or lack of improvement is normal in older patients with severe frailty undergoing TAVR or SAVR and also confirming the crucial role of frailty assessment in predicting various functional trajectories [7].

## 6. Limitations

The retrospectivity of the study and the limited size of the population examined are limitations of this single-center study. However, despite these limitations, the excellent characterization with a multidisciplinary approach means that our study presents some important conclusions that open the way for studies with larger populations. In addition, another limitation is that self-reported functional status was validated against objective measures; however, a geriatric frailty phone screening has been validated in several other studies.

## 7. Conclusions

Interventional treatment of severe symptomatic aortic stenosis in elderly patients with early degrees of frailty has proven to be effective and appropriate in terms of survival and functional status even in a long-term follow-up. The FIMS score also showed its prognostic capacity after 6 years of follow-up in defining the survival of elderly patients treated with TAVR/SAVR. Finally, the functional status of elderly survivor patients is slightly worse when there is no clinical worsening of heart failure. In this population, the interventional treatment of aortic stenosis is confirmed as appropriate in terms of short- and long-term outcomes. 

## 8. New Perspectives

**Figure 4:**
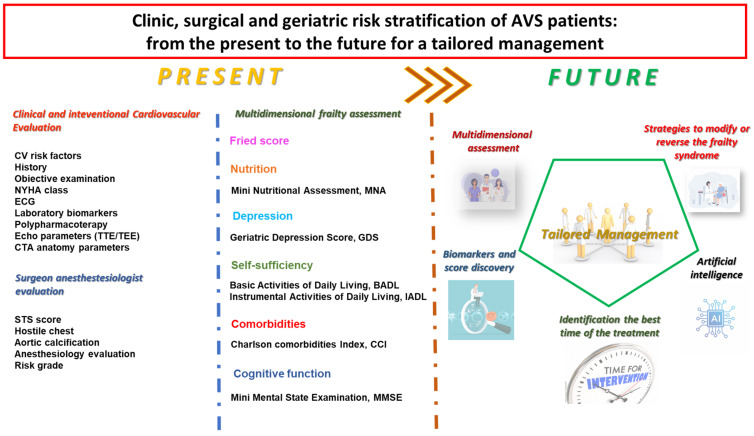
A graphic representation of the present and future strategies for tailored, appropriate management of aortic stenosis in elderly patients.

The goals of general clinical practice, and particularly in SA, come from the centrality of the patient. For patient-centered care, a multidisciplinary team must discuss the optimal timing of intervention and analyze the advantages and disadvantages related to various treatment options. As the care pathway of patients with AS is a challenge for physicians, defining new indicators and identifying preventive strategies are crucial for the advent of tailored diagnostic techniques and precision medicine. (Figure 4).

Thus, the decision-making on aortic valve replacement in patients aged 75 years could be addressed according to the follow points (Figure 4):

1. An accurate multidisciplinary (clinical–surgical, instrumental, functional geriatric) evaluation is required during cardiologic surveillance to identify the best time for an appropriate interventional treatment for AS, avoiding the occurrence of an acute heart failure event that worsens the frailty degree and outcomes.
2. To identify a frail status could lead to adopting strategies regarding physical activity and nutritional habits that may be beneficial to modify or reverse the frailty syndrome.
3. Should severe degenerative AS in elderly patients be operated on before symptom onset? Current guidelines for patients with asymptomatic severe aortic stenosis (AS) and preserved left ventricular ejection fraction recommend clinical surveillance every 6 to 12 months. New randomized trials, the largest thus far, are ongoing to assess the role of early intervention among patients with asymptomatic severe AS compared to clinical surveillance and the role of TAVR.
4. Symptoms are difficult to highlight in elderly patients with comorbidities. Therefore, the use of biomarkers and predictive scores can be useful in decision-making for early TAVR.
5. Artificial intelligence will certainly prove to be an objective tool for personalizing care.

## Figures and Tables

**Table 1:** Clinical and demographic data of survivor patients.

Variable	No Survivor (n = 65)	Survivor (n = 44)	*p*-Value
Age (yrs)	83.4 ± 5.8	83.1 ± 4.9	0.369
Male, n	29.7%	31%	0.813
Comorbidities			
Hypertension	85.9%	93.2%	0.239
Hypercholesterolemia	68.8%	79.5%	0.214
Diabetes	32.8%	38.6%	0.533
Smoking	26.6%	27.3%	0.959
Previous AMI	12.5%	20.5%	0.265
Previous stroke	12.5%	15.9%	0.615
Echo parameters			
PAPs	48.9 ± 11.8	43.3 ± 9.7	0.012
EF, %	55.4 ± 9.5	60.7 ± 5.9	0.001
mAVG, mmHg	42.2 ± 13.7	46.9 ± 9.4	0.053
STS score	6.1 ± 5.2	4.2 ± 2.4	0.0197
Clinical Chemistry data (FIMS Score)			
Haemoglobin (Hb), g/dL	11.9 ± 1.8	12.9 ± 3.2	0.003
Neutrophils, n/µL	5211 ±2010	4892 ± 1973	0.4
C-reactive protein (CRP), mg/dL	0.8 ± 1.7	0.5 ± 0.9	0.2
Creatine phosphokinase (CPK), IU/L	59.8 ± 31.1	81.7 ± 39.8	0.002
Creatinine, mg/dL	1.5 ± 1.4	1.02 ± 0.3	0.02
Troponin I, µg/L	0.03 ± 0.03	0.03 ± 0.03	0.67
High-Density Lipoprotein cholesterol (HDL), mg/dL	55 ± 17	58 ± 12	0.33
B-type natriuretic peptide (BNP), mg/L	685 ± 1153	310 ± 333	0.05
Albumin, g/dL	3.8 ± 0.4	4.2 ± 0.3	<0.0001
FIMS Score value	2.1 ± 1.7	1 ± 0.73	<0.001
Aortic valve treatment			
SAVR n(%)	3 (4.7)	5 (11.4)	0.001
TAVR n(%)	25 (39.1)	33 (75)	
BAV n(%)	21 (32.8)	6 (13.6)	
MT n(%)	15 (23.4)	0 (0)	
Physical frailty			0.001
Frail n (%)	26 (40.6)	4 (9.1)	
Early-frail n (%)	19 (29.7)	14 (31.8)	
Pre-frail n (%)	19 (29.7)	26 (59.1)	
Number of drugs	7.1 ± 2.7	6.3 ± 2.3	0.133
Geriatric features			
MMSE (0–30)	23.5 ± 5.7	26.4 ± 3.3	0.003
MNA (0–15)	9.7 ± 2.5	11.4 ± 1.7	0.001
BADL (0–6)	4.8 ± 1.8	5.6 ± 0.7	0.006
IADL (0–8)	5.5 ± 2.6	6.9 ± 1.5	0.001

Values are mean ± SD, n (%). AMI: acute myocardial infarction; PAPs: systolic pulmonary artery pressure; EF: ejection fraction; mAVG: mean aortic valve gradient; STS: Society of Thoracic Surgeons; SAVR: surgical aortic valve replacement; TAVR: transcatheter aortic valve replacement; BAV: balloon aortic valve; MT: medical therapy; MMSE: mini-mental state examination (score range, 1–30); MNA: Mini Nutritional Assessment (score range 0–14); BADL: basic activities of daily living, (score range, 0–6); IADL: instrumental activities of daily living (score range, 0–8).

**Table 2:** Geriatric functional assessment before and six years after a tailored interventional treatment based on frailty degree for severe aortic stenosis.

	Baseline			Follow-Up			
	Mean (SD)	Median IQR		Mean (SD)	Median IQR		*p*-Value
Charlson	3.63 (1.8)	2.0	3–4	4.54 (1.9)	3.2	4–5	<0.001
MNA	11.28 (1.7)	10.0	12–12.7	8.90 (2.7)	5.5	10.5–11	<0.001
BADL	5.66 (0.7)	6.0	6–6	4.50 (1.8)	3.2	5–6	<0.001
IADL	7.09 (1.2)	6.0	8–8	4.47 (3.3)	0.2	5–8	<0.001

MNA: Mini Nutritional Assessment; BADL: basic activities of daily living, IADL: instrumental activities of daily living.

## Data Availability

The dataset of this study is available from the corresponding author upon reasonable request.
